# Effects of clear aligners on the vertical position of the molar teeth and the vertical and sagittal relationships of the face: a preliminary retrospective before-after clinical trial

**DOI:** 10.1186/s12903-024-03972-w

**Published:** 2024-02-14

**Authors:** Mehrnaz Moradinejad, Ricky E. Harrell, Sayed Mohammad Mousavi, Minoo Alavi, Alireza Darvish Basseri, Arman Feiz, Hanie Daryanavard, Vahid Rakhshan

**Affiliations:** 1 Department of Orthodontics, School of Dentistry, Ahvaz Jundishapur University of Medical Sciences, Ahvaz, Iran; 2 Georgia School of Orthodontics, Atlanta, GA 30350 USA; 3 Oral and Maxillofacial Radiology, School of Dentistry, Ahvaz Jundishapur University of Medical Sciences, Ahvaz, Iran; 4 Formerly, Department of Anatomy, Dental School, Azad University of Medical Sciences, Tehran, Iran

**Keywords:** Clear Aligners, Adverse Effects, Predictability, Vertical Dimension, Molar Intrusion

## Abstract

**Introduction:**

Despite the popularity of clear aligners, their predictability has not been assessed adequately. Moreover, no study has investigated their effects on numerous dentomaxillary variables. Therefore, this study was conducted for the first time, assessing several new or controversial items. The aim of the study was to evaluate the effects of clear aligners on the vertical position of the molar teeth and the vertical and sagittal relationships of the face.

**Methods:**

This preliminary retrospective before-after non-randomized clinical trial was performed on 168 observations of 84 patients (33.60±9.28 years, 54 females) treated with 0.75mm Invisalign appliances. Pretreatment and posttreatment values were measured for: mandibular plane angle, occlusal plane angle, Y-Axis, ANB, facial angle, lower anterior facial height, overbite, and the distances of the molars from the palate and mandibular plane were measured. The alterations in parameters caused by treatment (delta values) were calculated for each measurement. Effects of treatment and some parameters on delta values were analyzed statistically (α=0.05).

**Results:**

Mean±SD of ΔMP-FH, ΔOP-FH, ΔY-Axis, ΔLAFH, ΔNPog-FH, ΔANB, ΔOverbite, ΔSNB, Δ6-PP, Δ7-PP, Δ6-MP, and Δ7-MP were respectively 0.11±1.61, 0.80±1.56, 0.15±1.18, 0.07±0.91, -0.22±1.25, 0.03±0.62, 0.04±1.15, -0.06±1.14, -0.36±0.94, -0.32±1.14, 0.19±0.96, 0.18±1.10. Only the alterations in OP-FH, 6-PP, and 7-PP were significant (*P*≤0.011). Age, sex, treatment duration, or pretreatment mandibular plane angle were not correlated with any delta values. However, the pretreatment occlusal plane angle was negatively correlated with ΔOP-FH and ΔY-Axis. Crowding was correlated negatively with ΔOP-FH and ΔY-Axis and positively with ΔNPog-FH. Overjet was negatively correlated with ΔANB and ΔOverbite (*P*≤0.035).

**Conclusions:**

Invisalign intruded first/second maxillary molars and increased the occlusal plane angle. Age, sex, and treatment duration were not correlated with post-treatment anatomic alterations.

## Introduction

There is a growing demand for appliances that are both more esthetic and more comfortable than traditional fixed appliances such as clear aligners [1–3]. The beautiful and transparent appearance, the removable nature of these appliances, and consequently the possibility of removing them in special social conditions and facilitating hygiene, are among the attractive advantages of treatment with these appliances, especially for adult patients [2–4]. In addition, some authors suggest a reduction in the duration of orthodontic treatment with these appliances compared to normal brackets [5]. Treatment with clear aligners has been a part of orthodontic treatment for decades, but especially since the introduction of Invisalign appliances in 1998, the use of this method has become a common approach in orthodontics [2, 3, 6].

Despite the advantages of using clear aligners (including aesthetics and comfort of the patient, ease of performing oral hygiene procedures, or lower risk of white spot lesions) and also despite the wide use of this method, there are only a few articles on the predictability of orthodontic movements using clear aligners [1–3, 7, 8]. The ability of clear aligners to achieve clinically acceptable results has been determined in some dental movements such as correcting the buccolingual inclination of the upper and lower incisors in mild to moderate degrees of malocclusion) [9].

Controlling the vertical dimension during treatment has always been a challenge in orthodontics [10]. The success of the treatment depends on the ability of the orthodontist to control the vertical movements of the teeth, because the extrusion of posterior teeth is the main cause of iatrogenic orthodontic side effects (including the backward rotation of the mandible) [10]. Also, adequate control of the vertical dimension is considered important in the successful correction of the anterior-posterior dimension [11]. For instance, therapeutic mechanics that extrude the posterior teeth lead to problems such as backward rotation of the mandible, opening the bite, and increasing the anterior height of the face; the backward rotation of the mandible subsequently worsens the class 2 molar relationship and overjet [12].

One of the problems that may affect the predictability of clinical results with the use of clear aligners is a slight posterior open bite that is seen in many patients who use these clear aligners; this situation is usually attributed to the possible intrusion of molars [3]. The mentioned issue can be the result of the thickness of clear aligners and the number of hours the patient has used them [4]. The amount of this intrusion may range from 0.25 to 0.5 mm, but there is no scientific evidence of the accuracy of these measurements [3]. Considering that there is no study to confirm this issue, some authors even deny the occurrence of the said problem [13]. As another limitation of clear aligners, it is difficult to correct extrusion, rotation, and overjet using them [14]. A recent systematic review also showed that clear aligners are effective in controlling anterior intrusion, but not effective in controlling anterior extrusion [15].

The literature has many large gaps: (1) So far, no study has been published on the relationship between vertical changes caused by clear aligners and the anteroposterior dimension of the face. (2) In addition, the vertical effect of clear aligners on the second molars (which itself plays an important role in controlling the vertical dimension of the face) has not been investigated yet. Considering the abovementioned importance of controlling the vertical dimension of the face in orthodontic treatment and the potential role of clear aligners in unwanted changes in this dimension, this research was conducted. Its aim was to compare cephalometric indices in lateral cephalograms before and after treatment of a group of patients who underwent orthodontic treatment with Invisalign with a thickness of 0.75 mm. The relationships between these changes with the age and sex of the patients and their pretreatment mandibular and occlusal plane angles were also assessed. The null hypotheses were a lack of any changes in cephalometric parameters between pretreatment and posttreatment cephalograms, as well as a lack of any associations between each of the parameters with demographics and occlusal or mandibular plane angles of the patients.

## Materials and methods

This retrospective before-after non-randomized clinical trial was done using 168 records of 84 patients treated in the Orthodontic Department of Georgia School of Orthodontics, Atlanta, GA, United States. Since all the radiographs were archival and no X-ray was emitted to any patients because of this study, and since no personal identifiers were to be collected, no harm would be imposed to any patients. Therefore, the research ethics were approved in accordance with the Helsinki declaration in 20/08/2022 (proposal number: U-01095, registration number: P.20.8.D.B.902). The study was commenced after the IRB approval. Since this study was carried out on anonymized and retrospectively taken human data, the need for informed consent to participate was waived by the Institutional Review Board of Ahvaz Jundishapur University of Medical Sciences, Ahvaz, Iran (ethics code: IR.AJUMS.REC.1401.189). The study ethics and its protocol were approved by the Institutional Review Board of Ahvaz Jundishapur University of Medical Sciences, Ahvaz, Iran (code: IR.AJUMS.REC.1401.189). All methods were performed in accordance with the relevant guidelines and regulations (including the Declaration of Helsinki); all experimental protocols were approved by the Institutional Review Board of Ahvaz Jundishapur University of Medical Sciences, Ahvaz, Iran.

### Sample size

The sample size was determined as All the available pre- and post-treatment lateral cephalograms of patients who met the eligibility criteria. After screening all the available 970 patients, 84 patients met the eligibility criteria and were included. The treatment of these patients had been carried out retrospectively between the years 2018 and 2021.

### Eligibility criteria

The inclusion criteria were patients undergone orthodontic treatment using 0.75mm Invisalign appliances (Align Technology, Tempe, Arizona, USA), with proper digital pretreatment and posttreatment lateral cephalograms, aged at least 18 years (the completion of growth), Class I dental relationships observed on dental casts, with the presence of complete eruption of all the permanent teeth (except the third molars), the presence of ClinCheck®, a lack of any ‘intrusion or extrusion or distalization or mesialization’ treatment plans the presence of pretreatment posterior teeth contacts (no posterior openbite), and the presence of non-extraction treatment plans. Excluded were patients aged less than 18 years, those having any diseases or syndromes affecting maxillofacial regions, any occlusal restorations of molars, inappropriate quality of pretreatment or posttreatment cephalograms, any incomplete documents, a lack of dental casts, the existence of any deepbite or open bite problems in the beginning of treatment, or a history of extraction treatment plans as well as any extractions of any other teeth such as 3rd molars during the course of treatment.

### Sample and Interventions

A total of 84 patients were included. Their treatment durations lasted between 6 and 46 months. The sample and treatment details are explained in the Results section.

### Measurements

All radiographs had been taken with two devices (Promax, SN: TDK252981, Planmeca, Helsinki, Finland). The digital cephalograms were traced using AudaxCeph software (Audax, Ljubljana, Slovenia). First, the landmarks were identified:

- Sella (S): The point representing the midpoint of the pituitary fossa.
- Nasion (N): The extreme anterior point on the fronto-nasal suture.
- Anterior Nasal Spine (ANS): The tip of the median, sharp bony process of the maxilla at the lower margin of the anterior nasal opening.
- Posterior Nasal Spine (PNS): The intersection of a continuation of the anterior wall of the pterygopalatine fossa and the floor of the nose, marking the dorsal limit of the maxilla.
- Gnathion (Gn): The most anteroinferior point on the contour on the bony chin symphysis.
- Menton (Me): The extreme inferior point of the mandibular symphysis.
- A-point (A): The deepest point on the curvature of the maxillary alveolar process.
- B-point (B): The deepest point on the curvature of the mandibular alveolar process.
- Pogonion (Pog): The extreme anterior point of the mandibular symphysis.
- Orbitale (Or): The deepest point on the infraorbital margin.
- Anatomic Porion (Po): The most superior and outer bony surface point of the external auditory meatus and can.
- Sella-Nasion-B Angle (SNB): The angle formed from by the intersection of Sella-Nasion and Nasion-B plane.
- A-Nasion-B Angle (ANB): The angle formed from the difference of SNA and SNB angle.

Afterwards, the following cephalometric measurements were estimated: the mandibular plane angle (with Frankfurt plane, in degrees), the occlusal plane angle (with Frankfurt plane, in degrees), Y-Axis (°), SNB (°), ANB (°), facial angle (in degrees), the lower anterior facial height ratio (%), and overbite (mm). For this purpose, the identified landmarks were checked jointly by four clinicians (an experienced orthodontist and three orthodontic residents/dentists) working together, and then the software calculated the measurements. Also, the same clinicians located the landmarks for the software to measure the following distances: the distance from the palatal plane to the mesiobuccal cusp of the upper first molar (mm), the distance from the mandibular plane to the mesiobuccal cusp of the lower first molar (mm), the distance from the palatal plane to the mesiobuccal cusp of the upper second molar (mm), and the distance from the mandibular plane to the mesiobuccal cusp of the lower second molar (mm). The definitions of the cephalometric variables are as follows:

- Over Bite (OB): The distance in mm between maxillary and mandibular incisal edges perpendicular to the occlusal plane.
- Lower anterior facial height ratio (%) (LAFH/TAFH): The ratio of the lower anterior facial height (ANS-Me) to total anterior facial height (N-Me).
- Facial Angle (FA, NPog-FH, Downs analysis): The angle formed from by the intersection of the Frankfort Horizontal and N-Pog.
- Mandibular Plane Angle (MP, Downs analysis): The angle formed by the intersection of the Frankfort Horizontal plane and the Mandibular plane.
- Y-Axis: The angle formed by the intersection of the Frankfort Horizontal plane and the S-Gn plane.
- The cant of the occlusal plane (OP, Downs analysis): The angle formed by the intersection of occlusal plane (the meeting point of the cusps of the first premolars and first molars) and the Frankfort Horizontal plane.
- The mandibular first molar’s position (L6-MP): The perpendicular distance in mm between the mesiobuccal cusp of the mandibular first molar and the mandibular plane.
- The mandibular second molar’s position (L7-MP): The perpendicular distance in mm between the mesiobuccal cusp of the mandibular second molar and the mandibular plane.
- The maxillary first molar’s position (U6-PP): The perpendicular distance in mm between the mesiobuccal cusp of the maxillary first molar and the palatal plane (ANS-PNS).
- The maxillary second molar’s position (U7-PP): The perpendicular distance in mm between the mesiobuccal cusp of the maxillary second molar and the palatal plane (ANS-PNS).

### Outcomes

The primary outcome of the study was the changes happened to the cephalometric variables as a result of treatment. This primary outcome was calculated as the posttreatment value of each parameter minus its pretreatment value; thus, positive delta values would indicate an increase in a given parameter after the course of the treatment, while negative delta values mean reduction in parameters.

The secondary outcomes were the associations between these anatomical alterations with patients’ demographics and pretreatment mandibular plane angle and pretreatment occlusal plane angle.

### Statistical analysis

A month after the initial measurements, all 12 cephalometric variables of 20 pretreatment and 20 posttreatment cephalograms of 20 randomly selected patients (a total of 40 lateral cephalograms) were re-analyzed jointly by both the main authors as detailed above. The intra-observer agreements were calculated for all 12 evaluated parameters, using the intraclass correlation coefficient (ICC) for single measures or average measures (the Cronbach’s Alpha).

Descriptive statistics and 95% confidence intervals (CIs) were computed for the pretreatment and posttreatment values as well as their differences (delta values) which were the main outcomes of this study. Data normality was assessed and confirmed using histograms, q-q plots, a Kolmogorov-Smirnov test, and a Shapiro-Wilk test. A Levene’s test was used to examine the equality of variances. For the baseline comparisons, an independent-samples t-test and a Fisher exact test were used to compare males and females in terms of baseline cephalometric measurements, orthodontic parameters, therapeutic parameters, and patients’ age. To assess the main null hypothesis (i.e., whether Invisalign altered any cephalometric parameters), the delta values (i.e., the alterations occurred during the course of treatment) were compared with the constant value zero using a one-sample t-test. To test the secondary null hypotheses, an independent-samples t-test was used to compare males and females in terms of the delta values (as a secondary outcome); moreover, correlations between age with the delta values were calculated using a Pearson correlation coefficient. Similarly, correlations between the delta values with the pretreatment mandibular plane angle and pretreatment occlusal plane angle were computed using the Pearson coefficient. All independent-samples t-tests were performed under the assumption of unequal variances. The software in use was SPSS 26 (IBM, Armonk, NY, USA). The level of significance was set at 0.05.

## Results

There was no missing data. There were 54 females and 31 males in the sample with an overall mean (and SD) age of 33.60 ± 9.28 years (minimum: 18, maximum: 56, 95% CI: 31.60 to 35.60). The mean age of females was 32.83 ± 8.80 years (minimum: 18, maximum: 54, 95% CI: 30.43 to 35.24); it was 34.94 ± 10.06 years for males (minimum: 18, maximum: 56, 95% CI: 31.25 to 38.62). According to the independent-samples t-test, the difference between ages of men and women was not significant (*P* = 0.317). Full details of the sample and treatment procedures are presented in Table 2. The one-sample t-test detected no significant difference between males and females in terms of any of the anatomical or therapeutic parameters (Table 1). Only 2 women and 3 men had crossbites; the sexes were not significantly different in this regard (Fisher, *P* = 0.353).

**Table 1:** Sample parameters, and the results of the t-test comparing males and females

**Variable**	**Sex**	**N**	**Mean**	**SD**	**95% CI**		**Min**	**Max**	***P***
**Maxillary Crowding (mm)**	**Female**	53	1.34	3.00	0.51	2.17	-6	6	0.398
	**Male**	31	1.90	2.82	0.87	2.94	-6	5	
	**Both**	84	1.55	2.93	0.91	2.18	-6	6	
**Mandibular Crowding (mm)**	**Female**	53	2.06	2.80	1.29	2.83	-5	6	0.302
	**Male**	31	2.68	2.36	1.81	3.54	-5	6	
	**Both**	84	2.29	2.65	1.71	2.86	-5	6	
**Overjet (mm)**	**Female**	53	2.49	0.93	2.23	2.75	0	4	0.204
	**Male**	31	2.23	0.88	1.90	2.55	1	4	
	**Both**	84	2.39	0.92	2.19	2.59	0	4	
**Treatment duration (months)**	**Female**	53	21.49	10.07	18.71	24.27	6	46	0.262
	**Male**	31	19.16	7.21	16.52	21.80	7	36	
	**Both**	84	20.63	9.15	18.65	22.62	6	46	
**Number of Main Aligners used for each patient**	**Female**	53	31.02	7.39	28.98	33.06	18	48	0.144
	**Male**	31	28.77	5.40	26.79	30.76	16	40	
	**Both**	84	30.19	6.78	28.72	31.66	16	48	
**Number of Refinement Courses after main treatment for each patient**	**Female**	53	1.79	1.56	1.36	2.22	0	6	0.714
	**Male**	31	1.68	1.01	1.31	2.05	0	4	
	**Both**	84	1.75	1.38	1.45	2.05	0	6	
**Number of aligners in the 1st Refinement course for each patient**	**Female**	47	20.26	6.83	18.25	22.26	6	37	0.515
	**Male**	29	19.17	7.28	16.40	21.94	6	37	
	**Both**	76	19.84	6.98	18.25	21.44	6	37	
**Number of aligners in the 2nd Refinement course for each patient**	**Female**	20	17.65	6.79	14.47	20.83	3	31	0.435
	**Male**	15	19.53	7.21	15.54	23.53	11	34	
	**Both**	35	18.46	6.93	16.08	20.84	3	34	
**Number of aligners in the 3rd Refinement course for each patient**	**Female**	12	18.08	6.95	13.67	22.50	10	33	0.548
	**Male**	6	16.00	6.45	9.23	22.77	9	27	
	**Both**	18	17.39	6.67	14.07	20.71	9	33	
**Number of aligners in the 4th Refinement course for each patient**	**Female**	9	12.67	6.98	7.30	18.03	4	25	0.554
	**Male**	2	9.50	0.71			9	10	
	**Both**	11	12.09	6.38	7.81	16.38	4	25	
**Aligner No. in the 5th Refinement**	**Female**	5	16.60	4.16	11.44	21.76	14	24	NA
**Aligner No. in the 6th Refinement**	**Female**	2	19.00	5.66			15	23	NA
**Number of All Aligners (Main aligners and Refinement aligners combined) for each patient**	**Female**	53	64.17	30.47	55.77	72.57	20	179	0.490
	**Male**	31	59.87	21.01	52.16	67.58	22	120	
	**Both**	84	62.58	27.30	56.66	68.51	20	179	

*NA* Not applicable, No men had any 5th or 6th refinement courses

### Intra-observer agreements

The intrarater agreements were excellent or perfect for all 12 variables (Cronbach’s alpha values ranging between 99.7% to 100%, all 12 *P* values = 0.00000).

### Baseline and posttreatment measurements

Descriptive statistics and 95% CIs for the pretreatment and posttreatment measurements are presented in Tables 2 and 3. Baseline comparisons showed no statistically significant difference between males and females (Table 3).

**Table 2:** Descriptive statistics and 95% CIs for the pretreatment and posttreatment values. The number of patients was 84

**Interval**	**Parameter**	**Mean**	**SD**	**Min**	**Q1**	**Med**	**Q3**	**Max**	**95% CI**	
**Baseline**	**MP-FH (°)**	23.87	5.49	9.20	20.58	23.60	27.33	42.80	22.68	25.06
	**OP-FH (°)**	7.38	3.71	-1.10	4.63	7.40	9.55	19.30	6.58	8.19
	**Y-Axis (°)**	60.14	3.42	50.80	58.05	59.85	61.88	68.10	59.40	60.88
	**LAFH/TAFH (%)**	56.11	2.37	50.60	54.48	56.40	58.00	61.40	55.60	56.63
	**NPog-FH (°)**	87.67	2.65	79.80	86.25	87.40	89.53	94.30	87.09	88.24
	**ANB (°)**	4.75	2.17	-1.40	3.55	4.70	6.33	10.60	4.28	5.22
	**Overbite (mm)**	2.22	1.38	-1.90	1.40	2.10	3.10	7.30	1.92	2.52
	**SNB (°)**	79.69	3.66	68.60	77.40	79.30	82.00	86.40	78.89	80.48
	**6-PP (mm)**	22.27	2.53	17.30	20.78	22.05	23.93	29.60	21.72	22.82
	**7-PP (mm)**	19.97	2.51	14.30	18.38	19.95	21.55	26.10	19.43	20.52
	**6-MP (mm)**	31.49	3.37	23.40	29.28	31.25	34.03	38.60	30.76	32.22
	**7-MP (mm)**	29.10	3.28	21.10	26.88	28.90	31.60	35.70	28.39	29.81
**Post-treatment**	**MP-FH (°)**	23.98	5.66	8.90	20.20	23.20	27.53	42.80	22.76	25.21
	**OP-FH (°)**	8.18	3.54	0.70	5.50	8.30	10.53	17.50	7.42	8.95
	**Y-Axis (°)**	60.29	3.32	52.00	58.38	60.30	62.03	68.10	59.57	61.01
	**LAFH/TAFH (%)**	56.19	2.53	50.00	54.18	56.55	58.00	60.60	55.64	56.73
	**NPog-FH (°)**	87.45	2.61	81.60	86.08	87.45	88.80	94.10	86.88	88.02
	**ANB (°)**	4.79	2.15	-0.40	3.48	5.00	6.13	10.00	4.32	5.25
	**Overbite (mm)**	2.26	1.15	-0.60	1.70	2.20	2.70	7.90	2.01	2.51
	**SNB (°)**	79.63	3.76	68.20	77.40	79.35	81.90	88.40	78.81	80.44
	**6-PP (mm)**	21.91	2.59	16.00	19.93	21.50	23.43	29.10	21.35	22.47
	**7-PP (mm)**	19.65	2.57	14.50	17.68	19.40	21.35	26.00	19.09	20.21
	**6-MP (mm)**	31.68	3.28	23.40	29.30	31.15	34.38	39.50	30.97	32.39
	**7-MP (mm)**	29.28	3.05	21.80	27.10	29.00	31.15	36.70	28.62	29.94

*SD* standard deviation, *Min* minimum, *Q1* first quartile, *Med* median, *Q3* third quartile, *Max* maximum, *CI* confidence interval

**Table 3:** Descriptive statistics and 95% CIs for the Baseline data in females and males, as well as their comparisons using the independent-samples t-test

**Parameter**	**Sex**	**N**	**Mean**	**SD**	**95% CI**		**Min**	**Max**	***P***
**MP-FH (°)**	**Female**	53	23.91	4.99	22.53	25.28	13.70	34.80	0.943
	**Male**	31	23.82	6.34	21.49	26.14	9.20	42.80	
**OP-FH (°)**	**Female**	53	7.64	3.88	6.57	8.71	0.40	19.30	0.411
	**Male**	31	6.95	3.41	5.70	8.19	-1.10	13.60	
**Y-Axis (°)**	**Female**	53	60.33	3.27	59.43	61.23	51.80	68.10	0.512
	**Male**	31	59.82	3.68	58.47	61.17	50.80	68.00	
**LAFH/TAFH (%)**	**Female**	53	56.03	2.43	55.36	56.70	50.60	61.40	0.680
	**Male**	31	56.25	2.31	55.40	57.10	52.30	59.90	
**NPog-FH (°)**	**Female**	53	87.45	2.61	86.73	88.17	79.80	92.30	0.340
	**Male**	31	88.03	2.73	87.03	89.03	83.10	94.30	
**ANB (°)**	**Female**	53	4.82	2.13	4.23	5.40	0.10	10.60	0.710
	**Male**	31	4.64	2.26	3.81	5.47	-1.40	8.30	
**Overbite (mm)**	**Female**	53	2.12	1.17	1.80	2.44	0.10	4.60	0.379
	**Male**	31	2.40	1.69	1.78	3.02	-1.90	7.30	
**SNB (°)**	**Female**	53	79.28	3.40	78.34	80.22	69.60	86.40	0.182
	**Male**	31	80.39	4.03	78.91	81.86	68.60	85.90	
**6-PP (mm)**	**Female**	53	22.01	2.26	21.38	22.63	17.50	27.10	0.218
	**Male**	31	22.72	2.92	21.65	23.79	17.30	29.60	
**7-PP (mm)**	**Female**	53	19.68	2.33	19.03	20.32	14.90	24.60	0.157
	**Male**	31	20.48	2.75	19.47	21.49	14.30	26.10	
**6-MP (mm)**	**Female**	53	31.40	3.20	30.52	32.28	23.40	38.10	0.759
	**Male**	31	31.64	3.68	30.29	32.98	25.20	38.60	
**7-MP (mm)**	**Female**	53	29.04	3.27	28.14	29.94	21.10	35.70	0.830
	**Male**	31	29.20	3.36	27.97	30.43	23.10	35.20	

*SD* standard deviation, *CI* confidence interval, *Min* minimum, *Max* maximum

Significant *P* values in bold font

### Primary outcomes

**Fig. 1:**
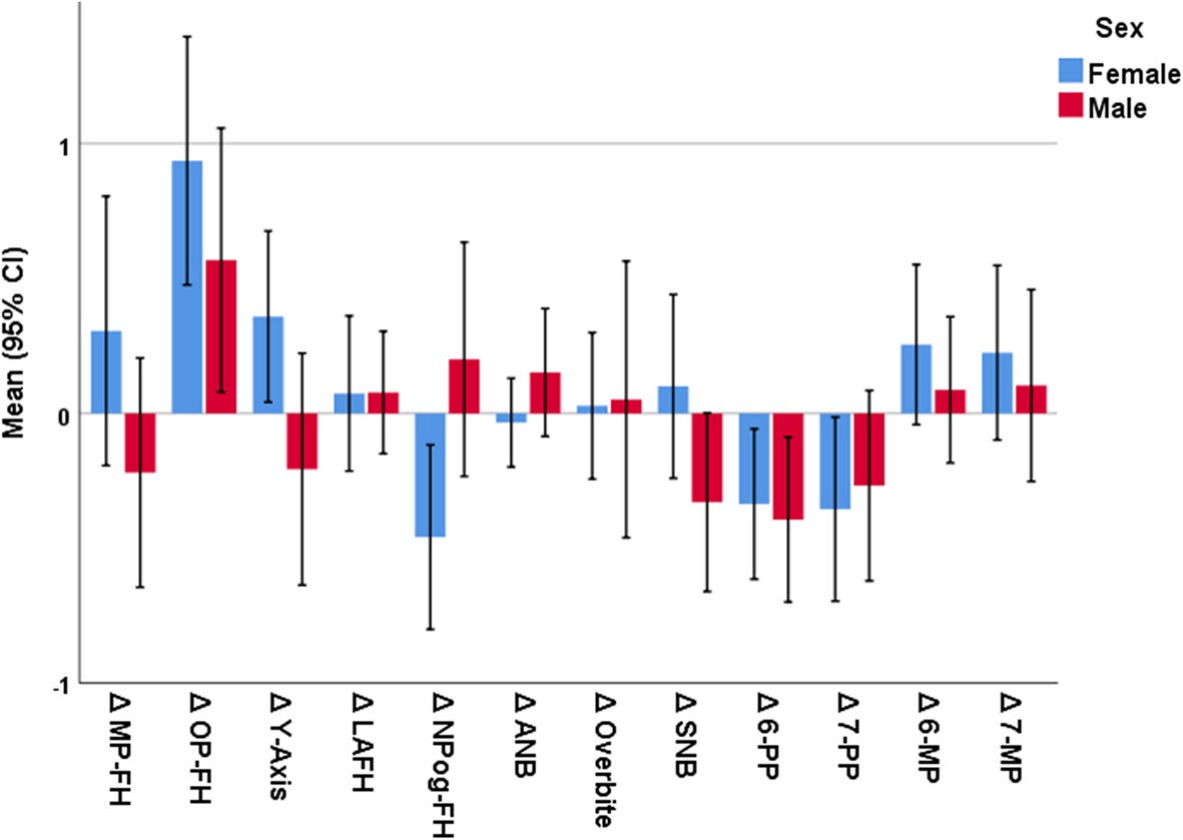
Mean (95% CI) for the extents of anatomical changes observed over the course of treatment. Positive delta values show an increase over time, while negative values indicate decreases

**Fig. 2:**
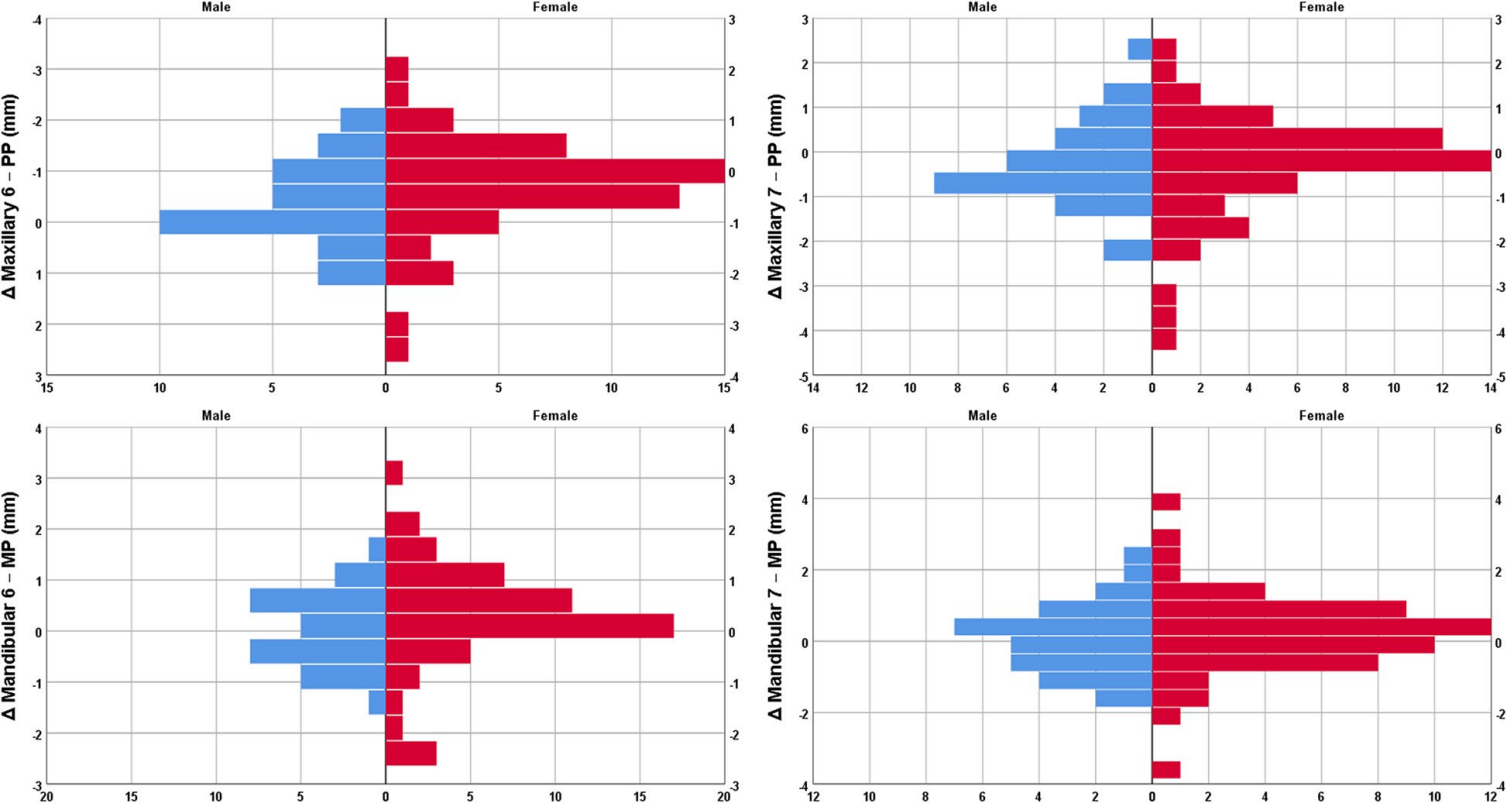
Histograms showing the numbers of male patients (blue) and female patients (red) with intrusions or extrusions of the molar teeth, as indicated by delta values. PP, palatal plane; MP, mandibular plane. Positive delta values show an increase over time, while negative values indicate decreases

The one-sample t-test showed that the occlusal plane angle increased significantly after the treatment, while 6-PP (the distance between the upper first molar and the palatal plane) and 7-PP (the distance between the upper second molar and the palatal plane) significantly decreased after the treatment (*P* ≤ 0.01, Table 4, Figs. 1 and 2). The number and percent of cases showing some extent of intrusion for the upper first molar were 53 (63.1%). These were 49 patients (58.3%) for the upper second molar, 30 patients (35.7%) for the lower first molar, and 34 (40.5%) for the lower second molar (Fig. 2).

**Table 4:** Primary Outcomes: Descriptive statistics and 95% CIs for the delta values (or the extents of changes in the cephalometric parameters during the treatment, *n* = 84). The *P* values are calculated by comparing the extents of change (delta values) with the value zero, using the one-sample t-test.

**Parameter**	**Mean**	**SD**	**Min**	**Q1**	**Med**	**Q3**	**Max**	**95% CI**		***P***
**Δ MP-FH (°)**	0.11	1.61	-3.60	-0.70	0.15	0.90	7.40	-0.24	0.46	0.52672
**Δ OP-FH (°)**	0.80	1.56	-2.50	-0.10	0.85	1.83	4.70	0.46	1.14	**0.00001**
**Δ Y-Axis (°)**	0.15	1.18	-2.80	-0.60	0.20	0.90	2.90	-0.11	0.41	0.24883
**Δ LAFH/TAFH (%)**	0.07	0.91	-2.70	-0.40	0.10	0.42	3.40	-0.12	0.27	0.44987
**Δ NPog-FH (°)**	-0.22	1.25	-3.30	-0.82	-0.30	0.50	2.60	-0.49	0.06	0.11880
**Δ ANB (°)**	0.03	0.62	-1.90	-0.43	0.10	0.40	1.60	-0.10	0.17	0.60996
**Δ Overbite (mm)**	0.04	1.15	-3.60	-0.50	0.00	0.43	5.80	-0.21	0.29	0.76853
**Δ SNB (°)**	-0.06	1.14	-2.30	-0.83	-0.10	0.70	3.90	-0.31	0.19	0.63982
**Δ 6-PP (mm)**	-0.36	0.94	-3.50	-0.80	-0.20	0.20	1.80	-0.56	-0.15	**0.00083**
**Δ 7-PP (mm)**	-0.32	1.14	-4.20	-0.80	-0.25	0.30	2.30	-0.57	-0.08	**0.01102**
**Δ 6-MP (mm)**	0.19	0.96	-2.40	-0.30	0.20	0.73	3.20	-0.02	0.40	0.07024
**Δ 7-MP (mm)**	0.18	1.10	-3.60	-0.43	0.20	0.80	3.70	-0.06	0.42	0.13734

*SD* standard deviation, *Min* minimum, *Q1* first quartile, *Med* median, *Q3* third quartile, *Max* maximum, *CI* confidence interval

Significant *P* values in bold font. Positive delta values show an increase in parameters over time, while negative values indicate decreases as a result of treatment

### Secondary outcomes

There was no significant difference between the sexes in terms of the alterations in any of the parameters except for Y-axis which decreased in men but increased in women as well as NPog-FH which increased in men but decreased in women (Table 5, Figs. 1 and 2). There was no significant correlation between patients’ ages and any of their anatomical alterations caused by the treatment (Table 6). Similarly, the pretreatment mandibular plane angle was not correlated with any of the alterations. However, the pretreatment occlusal plane angle was negatively correlated with Δ OP-FH and Δ Y-Axis (Table 6). Both maxillary and mandibular crowding extents were correlated negatively with Δ OP-FH and Δ Y-Axis (marginally significant in the case of maxillary crowding against Y-Axis) and positively with Δ NPog-FH (Table 6). Overjet extents were negatively correlated with Δ ANB and Δ Overbite (Table 6). Treatment duration was not correlated with any anatomic alterations (Table 6).

**Table 5:** Secondary Outcomes: Descriptive statistics and 95% CIs for the alterations in cephalometric parameters of females and males. The *P* value is calculated using the independent-samples t-test

**Parameter**	**Sex**	**N**	**Mean**	**SD**	**95% CI**		**Min**	**Max**	***P***
**Δ MP-FH (°)**	**Female**	53	0.31	1.81	-0.19	0.80	-3.60	7.40	0.151
	**Male**	31	-0.22	1.16	-0.64	0.21	-3.30	1.90	
**Δ OP-FH (°)**	**Female**	53	0.94	1.67	0.48	1.40	-2.50	4.70	0.298
	**Male**	31	0.57	1.33	0.08	1.06	-2.30	2.50	
**Δ Y-Axis (°)**	**Female**	53	0.36	1.15	0.04	0.68	-2.50	2.90	**0.034**
	**Male**	31	-0.21	1.17	-0.64	0.22	-2.80	2.00	
**Δ LAFH/TAFH (%)**	**Female**	53	0.07	1.04	-0.21	0.36	-2.70	3.40	0.985
	**Male**	31	0.08	0.62	-0.15	0.30	-1.60	1.30	
**Δ NPog-FH (°)**	**Female**	53	-0.46	1.24	-0.80	-0.12	-3.30	2.60	**0.019**
	**Male**	31	0.20	1.18	-0.23	0.63	-1.90	2.60	
**Δ ANB (°)**	**Female**	53	-0.03	0.60	-0.20	0.13	-1.90	1.60	0.186
	**Male**	31	0.15	0.65	-0.09	0.39	-1.40	1.40	
**Δ Overbite (mm)**	**Female**	53	0.03	0.98	-0.24	0.30	-3.60	1.80	0.929
	**Male**	31	0.05	1.40	-0.46	0.56	-2.30	5.80	
**Δ SNB (°)**	**Female**	53	0.10	1.24	-0.24	0.44	-2.30	3.90	0.096
	**Male**	31	-0.33	0.90	-0.66	0.00	-2.20	1.90	
**Δ 6-PP (mm)**	**Female**	53	-0.34	1.01	-0.61	-0.06	-3.50	1.80	0.789
	**Male**	31	-0.39	0.83	-0.70	-0.09	-2.20	1.10	
**Δ 7-PP (mm)**	**Female**	53	-0.35	1.24	-0.70	-0.01	-4.20	2.10	0.737
	**Male**	31	-0.27	0.96	-0.62	0.08	-2.20	2.30	
**Δ 6-MP (mm)**	**Female**	53	0.25	1.08	-0.04	0.55	-2.40	3.20	0.445
	**Male**	31	0.09	0.74	-0.18	0.36	-1.40	1.80	
**Δ 7-MP (mm)**	**Female**	53	0.22	1.17	-0.10	0.55	-3.60	3.70	0.628
	**Male**	31	0.10	0.97	-0.25	0.46	-1.50	2.50	

*SD* standard deviation, *CI* confidence interval, *Min* minimum, *Max* maximum

Significant *P* values in bold font. Positive delta values show an increase in parameters over time, while negative values indicate decreases as a result of treatment

**Table 6:** Secondary outcomes: The results of the Pearson correlation coefficient calculated between the delta values (alterations caused by treatment) with age, treatment durations, and orthodontic parameters. Each *n* = 84

**Variable**		**ΔMP-FH**	**ΔOP-FH**	**ΔY-Axis**	**ΔLAFH**	**ΔNPog-FH**	**ΔANB**	**ΔOB**	**ΔSNB**	**Δ6-PP**	**Δ7-PP**	**Δ6-MP**	**Δ7-MP**
**Age (years)**	**R**	-0.082	-0.005	-0.160	0.044	0.165	0.139	0.083	0.063	-0.073	-0.084	-0.108	-0.019
	***P***	0.460	0.960	0.147	0.694	0.135	0.207	0.452	0.567	0.509	0.447	0.326	0.865
**Occlusal Plane Angle (°)**	**R**	-0.117	**-0.312**	**-0.233**	0.125	0.120	0.162	-0.043	-0.044	0.064	0.070	0.073	0.063
	***P***	0.288	**0.004**	**0.033**	0.256	0.276	0.141	0.699	0.692	0.564	0.524	0.509	0.567
**Mandibular Line Angle (°)**	**R**	-0.043	-0.102	-0.113	0.119	-0.019	0.018	0.070	-0.021	0.011	-0.010	0.000	0.017
	***P***	0.698	0.358	0.306	0.282	0.864	0.869	0.528	0.849	0.923	0.930	0.996	0.877
**Maxillary Crowding (mm)**	**R**	-0.154	**-0.230**	-0.200	0.023	**0.230**	-0.158	0.084	0.048	0.035	0.011	-0.043	-0.108
	***P***	0.162	**0.035**	0.068	0.836	**0.035**	0.152	0.447	0.667	0.749	0.921	0.698	0.326
**Mandibular Crowding (mm)**	**R**	-0.126	**-0.277**	**-0.238**	0.019	**0.296**	-0.027	0.060	0.026	0.027	-0.007	-0.003	-0.002
	***P***	0.254	**0.011**	**0.029**	0.863	**0.006**	0.804	0.587	0.817	0.805	0.952	0.979	0.988
**Overjet (mm)**	**R**	0.030	-0.005	-0.057	0.145	0.082	**-0.281**	**-0.238**	0.088	-0.045	-0.012	-0.051	-0.077
	***P***	0.786	0.964	0.606	0.187	0.460	**0.010**	**0.029**	0.427	0.686	0.913	0.644	0.487
**Treatment duration (months)**	**R**	0.048	0.147	0.041	-0.036	-0.028	-0.001	-0.037	0.052	0.085	0.041	0.072	0.103
	***P***	0.662	0.181	0.708	0.745	0.802	0.995	0.738	0.636	0.444	0.713	0.513	0.350

*R* correlation coefficient

## Discussion

In the present study, the changes in the vertical and anteroposterior angles of the face (except the occlusal plane) were not significant. It can be expected that following a change in the position of the teeth in different dimensions, changes will also be made to the face appearance [3]. Similar to earlier research, the patients in the current study were also selected from adult patients to eliminate the effects of tooth growth and changes in the vertical and sagittal dimensions of the face following growth as a confounding factor [3, 10]. Furthermore, patients who received restorative treatment (during the treatment on the occlusal surface of the posterior teeth) were also excluded from the study, because this factor can cause errors in the accurate examination of the vertical position of the posterior teeth. In addition, patients who needed distalization or mesialization of molars were also excluded from this study, because changing the sagittal position of molars can affect the vertical dimension and consequently the horizontal dimension of the face.

In the current study, despite not planning for any intrusions, our results indicated a significant effect of these appliances on the vertical position of the upper molars and the significant intrusion of the upper first and second molars (for an average of 0.3 mm) following the use of clear aligners. These intrusions occurred in more than 60% of people. Talens-Cogollos et al. [3] as well observed some degrees of molar intrusion (for an average of 0.94 mm) in 74.2% of patients after treatment with aligners. Womack et al. [16] estimated the amount of this intrusion to be around 0.25 to 0.5 mm. This posterior intrusion was also confirmed in other studies [17, 18]. The thickness of clear aligner can play a role in such posterior intrusions [4]. Additionally, the long duration of using these aligners during the day can also contribute to this intrusion, because continuous forces can lead to intrusion. The latter is inevitable since it is recommended to use the aligners at least 22 hours a day to have the desired effects [4]. In the present study, although the intrusion of the mandibular molars was observed in about 40% of patients, it was not notable in terms of severity and prevalence compared to the amount of intrusion happened to the upper molar teeth. Talens-Cogollos et al. [3] as well witnessed molar intrusion in both arches in 25.9% of patients. The difference in bone density in the posterior region of the mandible and maxilla can justify easier intrusion in the posterior region of the maxilla. According to the bone density classification, the bone in the posterior region of the maxilla is often of D4 type, which represents fine trabecular bone; whereas, In the posterior mandible, the bone is of type D3 or in some cases D2, which indicates porous cortical bone [19]. In agreement with our finding, Suh et al. [20] as well did not observed significant intrusions in the posterior mandible, even despite planning for lower molar intrusion for 0.5 to 0.6 mm. However, Moshiri et al. [21] stated that aligners cannot intrude posterior teeth without any planned design. According to them, neither the aligner thickness is adequate to overcome the freeway space nor the bite force lasts for a sufficient period of time to be able to exert a significant intrusion.

In this study, there was no significant change in the vertical and horizontal positions of the mandible after treatment with aligners. Although molar intrusion is expected to decrease the mandibular plane angle, increase the chin prominence, decrease inter-labial gap and facial convexity, increase bite and decrease overjet and forward movement of lower lip, if the amount of intrusion is small or if it is followed by the extrusion of mandibular molars, the above changes may not be seen significantly [22]. In the study of Talens-Cogollos et al. [3], no significant changes in the mandibular plane were observed in any of the patients at the end of the treatment, which was attributed to the small amount of posterior intrusion. Suh et al. [20] as well reported no significant changes in the vertical dimension, which can be related to the high number of hyperdivergent patients in their study. In other words, it might be said that clear aligners control the vertical dimension of the face rather than reducing it [20]. In addition, in two studies, treatment with clear aligners in open bite patients was associated with dental movements and not skeletal changes [21, 23].

The relationship between the mandibular plane angle (in fact, the relationship between the vertical dimension and muscle force) and the vertical changes of the molar teeth was statistically insignificant in the present study. However, in a past study, significant rates of unwanted posterior intrusion after treatment with clear aligners was observed in patients with facial brachycephalic patterns and short faces [24]. Some evidence showed that different mandibular plan angles registered different force bites [25]. The difference can be due to different methodologies such as treatment protocols or durations as well as the number of boys or girls and the number of cases with different vertical patterns of growth. Additionally, the thickness and types of the clear aligners may matter. More studies are needed to verify the results.

Dental movements are expected to be greater in younger patients and in women due to their lower bone densities [26]. Nevertheless, in our study no significant relationship between age and dental or skeletal changes was observed. We also did not observe any links between treatment duration and anatomic alterations. Perhaps, this might be explained to some extent by noting that after some time, the treatment effect may slow down, reaching a plateau. These two negative results need future investigations.

This study was limited by some factors. Although we screened all the available patients, still a larger sample size collected from two or more centers would improve the reliability fo results. However, it should be noted that the available few studies in this regard were all smaller than this study with sizes as small as for example 24 patients [1] or 42 patients [8]. Another limitation of this study was its retrospective nature; it was not possible ethically to expose patients to the hazardous and ionizing X-ray radiation for research purposes. Therefore, we were limited to using archival radiographs. Finally, the generalizability of the results was limited to only one brand and thickness of clear aligners. Therefore, future studies are warranted to assess other types or brands of clear aligners.

## Conclusions

Invisalign intruded both the first and second maxillary molars and increased the occlusal plane angle. None of the alterations were affected by age, sex, or treatment duration. Similarly, the pretreatment mandibular plane angle was not correlated with any of the changes. However, the pretreatment occlusal plane angle was negatively correlated with modifications in the occlusal plane angle or Y-Axis. Crowding was negatively correlated with alterations in the occlusal plane angle or Y-Axis and positively correlated with NPog-FH changes. Overjet was negatively correlated with post-treatment changes in ANB and overbite.

## Data Availability

The data are available from the corresponding author upon request.
